# Clustering and genetic differentiation of the normocyte binding protein (nbpxa) of *Plasmodium knowlesi* clinical isolates from Peninsular Malaysia and Malaysia Borneo

**DOI:** 10.1186/s12936-016-1294-6

**Published:** 2016-04-26

**Authors:** Md Atique Ahmed, Mun Yik Fong, Yee Ling Lau, Ruhani Yusof

**Affiliations:** Department of Parasitology, Faculty of Medicine, University of Malaya, 50603 Kuala Lumpur, Malaysia; Tropical Infectious Diseases Research and Education Centre (TIDREC), University of Malaya, 50603 Kuala Lumpur, Malaysia

**Keywords:** *Plasmodium knowlesi*, Normocyte binding protein, Types, Natural selection

## Abstract

**Background:**

The zoonotic malaria parasite *Plasmodium knowlesi* has become an emerging threat to South East Asian countries particular in Malaysia. A recent study from Sarawak (Malaysian Borneo) discovered two distinct normocyte binding protein xa (Pknbpxa) types of *P. knowlesi*. In the present study, the *Pknbpxa* of clinical isolates from Peninsular Malaysia and Sabah (Malaysian Borneo) were investigated for the presence of *Pknbpxa* types and natural selection force acting on the gene.

**Method:**

Blood samples were collected from 47 clinical samples from Peninsular Malaysia (n = 35) and Sabah (Malaysian Borneo, n = 12) were used in the study. The *Pknbpxa* gene was successfully amplified and directly sequenced from 38 of the samples (n = 31, Peninsular Malaysia and n = 7, Sabah, Malaysian Borneo). The *Pknbpxa* sequences of *P. knowlesi* isolates from Sarawak (Malaysian Borneo) were retrieved from GenBank and included in the analysis. Polymorphism, genetic diversity and natural selection of Pknbpxa sequences were analysed using DNAsp v 5.10, MEGA5. Phylogentics of Pknbpxa sequences was analysed using MrBayes v3.2 and Splits Tree v4.13.1. The pairwise *F*_*ST*_ indices were used to determine the genetic differentiation between the Pknbpxa types and was calculated using Arlequin 3.5.1.3.

**Results:**

Analyses of the sequences revealed *Pknbpxa* dimorphism throughout Malaysia indicating co-existence of the two types (Type-1 and Type-2) of *Pknbpxa*. More importantly, a third type (Type 3) closely related to Type 2 *Pknbpxa* was also detected. This third type was found only in the isolates originating from Peninsular Malaysia. Negative natural selection was observed, suggesting functional constrains within the *Pknbpxa* types.

**Conclusions:**

This study revealed the existence of three *Pknbpxa* types in Malaysia. Types 1 and 2 were found not only in Malaysian Borneo (Sarawak and Sabah) but also in Peninsular Malaysia. A third type which was specific only to samples originating from Peninsular Malaysia was discovered. Further genetic studies with a larger sample size will be necessary to determine whether natural selection is driving this genetic differentiation and geographical separation.

## Background

*Plasmodium knowlesi* is a natural malaria parasite of long-tailed and pig-tailed macaques. Since 2004, natural human infections due to this parasite have been increasingly reported from almost all Southeast Asian countries with highest cases being reported from Malaysia [1–3]. Among all the malaria cases reported in Malaysia, *P. knowlesi* infections are the highest. At least 10 % of the patients suffered severe or complicated malaria with a case fatality rate of 1–2 % [4, 5]. *P. knowlesi* infection has been recognized as zoonotic as human to human transmission has not yet been found in natural infection [6]. With its 24-h erythrocytic cycle, parasite invasion occurs daily in *P. knowlesi* infection. The infection may lead to severe disease that involves renal failure, liver dysfunction and respiratory distress [7, 8].

The reticulocytes binding protein (RBP) family has been identified in all *Plasmodium* species and has been proved to play a vital role in merozoite invasion into red blood cells (RBCs) [9–11]. A recent study showed that particular alleles of RBP gene family of *P. knowlesi* (*nbpxa* and *nbpxb*) were linked to high parasitaemia and disease severity in human infections [12]. Of particular interest, the normocyte binding protein xa (*Pknbpxa*) was found to be dimorphic. One of the dimorphic forms, KH195, was associated with disease severity, suggesting potential link between invasion phenotypes, parasitaemia and virulence [12]. Further study revealed that this dimorphism was present not only in the *Pknbpxa* gene but across the complete genomes of six *P. knowlesi* isolates from Sarawak, Malaysian Borneo, thus indicating the presence of two types of the parasite in that region [13]. A microsatellite-based study also identified two divergent parasite populations in Malaysia [14]. In addition to this, a recent genome study on clinical isolates indicated two major sympatric sub-clusters originating from Sarawak, Malaysian Borneo and a third cluster comprising parasite lines originating from Peninsular Malaysia and the Philippines [15].

In order to determine whether the two *P. knowlesi* types are present in other regions of Malaysia, the polymorphic RBC binding domain of the *Pknbpxa* gene (885 bp) of clinical samples obtained from Peninsular Malaysia and Sabah (Malaysian Borneo) was sequenced and analysed. Previously reported *Pknbpxa* sequences were also included in the study. Stringent phylogenetic and sequence analysis tools were used to identify *Pknbpxa* dimorphism, associating SNPs and natural selection within the *Pknbpxa* types.

## Methods

### Blood samples

A total of 47 blood samples were used in this study. Thirty-five blood samples were collected from patients infected with *P. knowlesi* from the University of Malaya Medical Centre (UMMC), Kuala Lumpur, Malaysia between July 2008 and July 2014. Twelve blood samples obtained from a previous study in Sabah [2] was also included in this study. The blood samples were collected by trained nurses in the infectious disease ward of UMMC. All patients exhibited clinical symptoms associated with malaria. Thin and thick blood smears were prepared from the patient’s blood for microscopic confirmation. Further diagnostic confirmation was done using nested polymerase chain reaction (PCR) [16] and BinaxNOW^®^ malaria rapid diagnostic test. Treatment was administered to patients tested positive for malaria, based on the guidelines of the Ministry of Health, Malaysia. Samples were selected at random with the only selection criterion being that they were single infection by *P. knowlesi.* Ethical clearance for this study was obtained from University of Malaya Medical Ethics Committee (Ref No. 817.18) and also by the Medical Research and Ethics Committee of the Malaysian Ministry of Health (Reference Number: KKM/NIHSEC/800/-2/2/2/P13-316). Informed verbal consent from the patient was obtained for use of the samples for diagnosis and research. Written consent was found to be unnecessary as verbal consent would be sufficient for the purpose of this study and patient details were noted down solely for record keeping. This consent procedure was approved by the University of Malaya Medical Centre Ethic Committee.

### Extraction of DNA

Genomic DNA was extracted from 100 µl blood sample by using QIAGEN blood and tissue extraction kit (Hilden, Germany) according to the manufacture’s instruction. DNA was analysed using *Plasmodium* genus and species specific nested PCR assays based on the *Plasmodium* small subunit ribosomal RNA (SSU rRNA) as described previously [16, 17].

### PCR and direct sequencing of *Pknbpxa*

The *Pknbpxa* gene was amplified and the amplicons were sequenced based on the modified protocol of a previous study [1]. Briefly, The primer pair PknbpxaF5 5′-AGGTGCAAGCTGGGAACAAG- 3′ and 7428R1 5′- GCCAAGTCCAAACTTTTCCC- 3′ was used to amplify part of the *Pknbpxa* with the following conditions: 2.0 ul DNA template, 0.4 U Phusion High-fidelity DNA polymerase (Thermo Scientific), 0.25 mM each primer, 500 uM each dNTP, 1× Phusion buffer (1.5 mM MgCl_2_) in 20 ml final volume. The cycling conditions were 98 °C for 30 s and then 38 cycles at 98 °C for 7 s, 64.8 °C for 20 s and 72 °C for 36 s, followed by a final extension at 72 °C for 10 min. PCR products were purified using the PCR DNA fragments extraction kit, (Geneaid, Biotech Ltd.) as per manufacturer’s instructions. Direct PCR sequencing was performed using the primer PknbpxaF11 5′-TAAGCGAATCGAATAAGCAGCAG-3′ with 4 ul BigDye Terminator v3.1 Cycle Sequencing (Applied Biosystems, Life Technologies). PCR product (34–36 ng) was included in 10 ul final volume reactions under the following conditions: 96 °C 20 s, 50 °C 15 s 60 °C 4 min for 35 cycles. The reactions were ethanol/sodium acetate precipitated and dried before sending to First BASE Pte Ltd (Malaysia) for sequencing.

### Analysis of *Pknbpxa* sequences

All raw sequences generated were analysed and trimmed using SeqMan software, Lasergene v 7.0 (DNASTAR). Sequences were aligned using the CLUSTAL-W program in MegAlign Lasergene v 7.0 (DNASTAR) and exported in FASTA format for polymorphic and phylogenetic analyses. For analysis, only sequences with unambiguous base calls were used. Mixed genotype infections (sequences with two calls at particular sites) were excluded. *Pknbpxa* sequences from Sarawak, Malaysian Borneo [12] [GenBank:KF186572, KF186571, KF186570, KF186568, KF186569] were included in the analysis along with the H-strain [GenBank:EU867791]. The *Pknbpxa* sequences were also retrieved from ten full genomes of *P. knowlesi* Sarawak isolates and full genomes of two laboratory strains from Peninsular Malaysia [GenBank:ERR274225, ERR274224, ERR366425, ERR274221, ERR366426, ERR274222, ERR985387, ERR985379, ERR985417, ERR985397, H(AW), Malayan] for inclusion in the analysis.

Sequence diversity (π), defined as the average number of nucleotide differences per site between two sequences within the sequences, was determined by DnaSP v5.10 software [18]. Parsimony informative sites and number of synonymous and non-synonymous substitutions within the *Pknbpxa* sequences was also determined by DnaSP v5.10 software. The rates of synonymous (dS) and non-synonymous (dN) mutations were estimated and compared by the Z-test (P < 0.05) in MEGA5 using the Nei and Gojobori’s method with the Jukes and Cantor (JC) correction and 1000 bootstrap replications [19, 20]. The McDonald and Kreitman (MK) test was performed with *Plasmodium cynomolgi RBP3* gene [GenBank:JQ422043] as an out group for testing natural selection acting within the genes using DnaSP v5.10 software.

### Phylogenetic analysis

Two phylogenetic methods were used to analyse the Pknbpxa gene sequences along with other reticulocyte binding ligand (RBL) genes from other *Plasmodium* species (*Plasmodium vivax* RBP1 &2 [GenBank:M88097, M88098], *Plasmodium yoelii* RBP2A [GenBank:XM_726167], *P. cynomolgi* RBP3 [GenBank:JQ422043 & JQ422050]).

### Bayesian phylogeny

Bayesian support (posterior probabilities) for the nodes was inferred through a Monte Carlo Markov chain model (MCMC) as implemented in Mr. Bayes [21], with 100,00,000 generations after a “burn-in” of 3000,000 generations (30 %). 6 (GRT) model was used for the phylogeny.

### Split decomposition graphs

Graphs were constructed using the computer program SplitsTree (version 4.0) [22]. Analysis was run using 1000 bootstrap replications.

### Population differentiation

ARLEQUIN software package version 3.5.1.3 was used to compute pairwise differences *F*_*ST*_ between Pknbpxa types with 10,100 permutations. *F*_*ST*_ is a comparison of the sum of genetic variability within and between populations based on the differences in allelic frequencies. *F*_*ST*_ values were interpreted as no differentiation (0), low (>0–0.05), moderate (0.05–0.15), and high (>0.15) genetic differentiation.

## Results

**Fig. 1:**
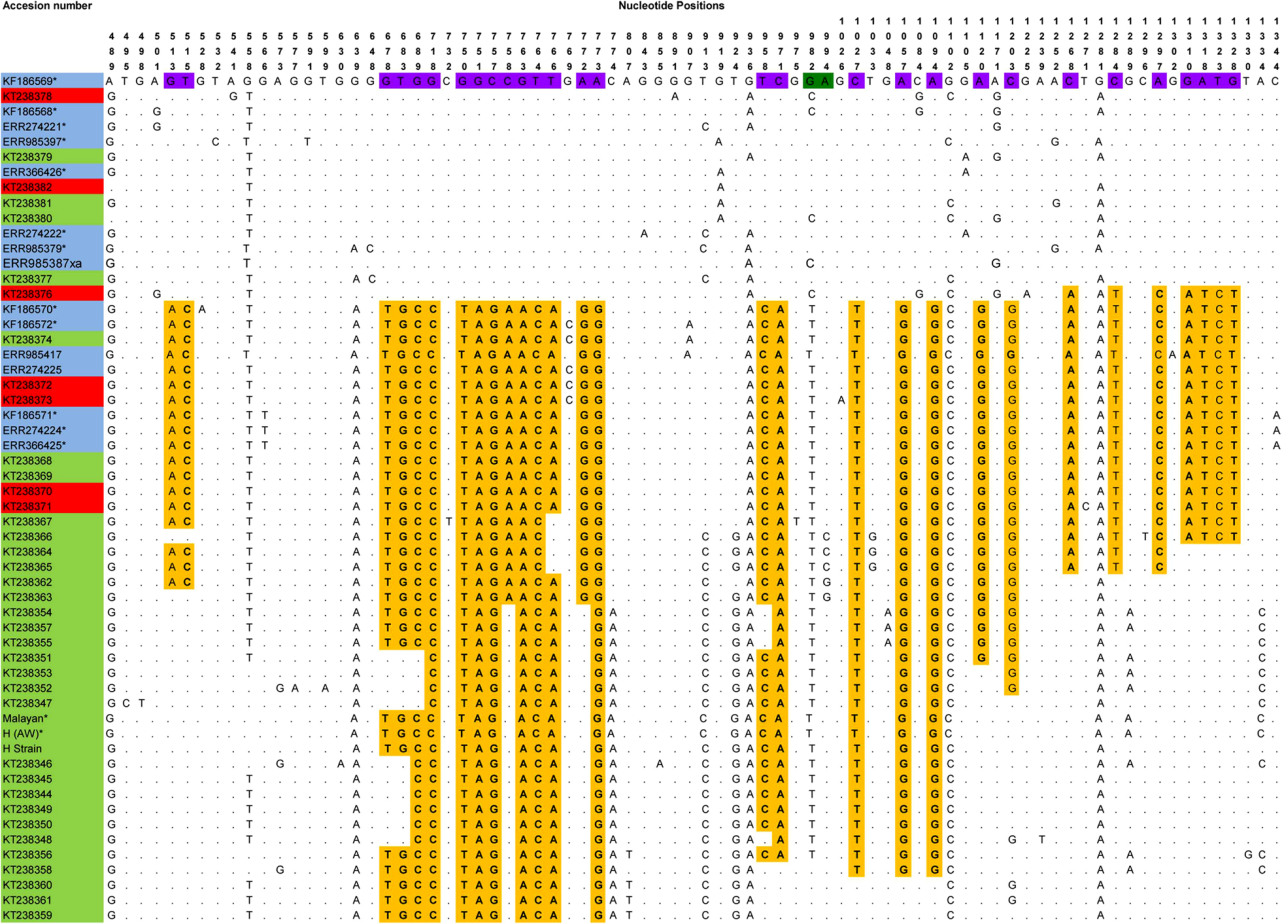
*Pknbpxa* polymorphisms and dimorphic residues. *Plasmodium knowlesi* nbpxa polymorphisms and the 29 core nucleotides making up the dimorphism. Dimorphic nucleotides are shaded *purple* and *yellow*. The non-synonymous SNPs are marked in *bold* and identical SNPs are marked as *dots*. SNPs with three variants are shaded in *dark green*. Accession numbers in *red*, *green* and *blue* indicate samples originating from Sabah, Peninsular Malaysia and Sarawak respectively. *Asterisk* indicates samples from previous study

**Fig. 2:**
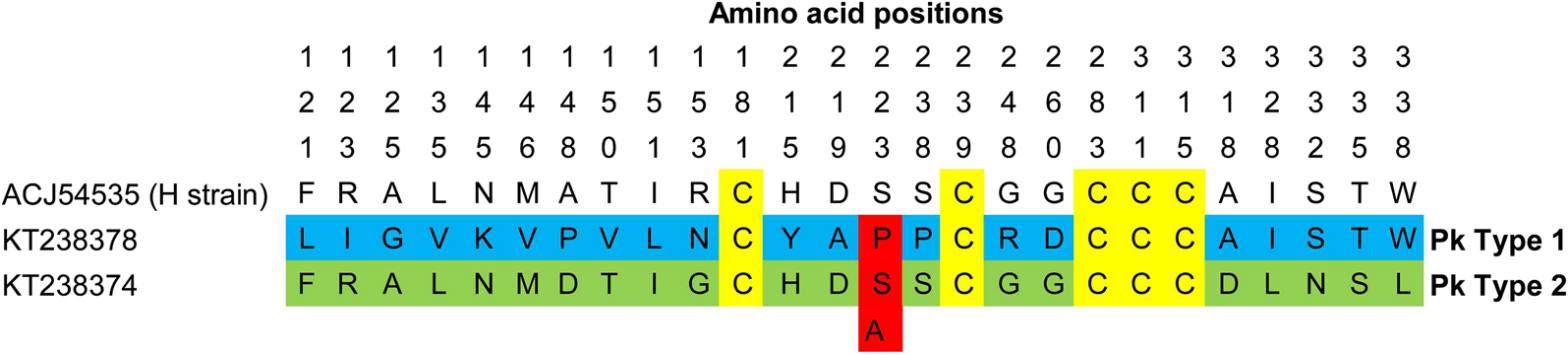
Detail amino acid sequence polymorphism in the *Pknbpxa* gene. Amino acid map highlighting the dimorphic residues (in *blue* and *green*) and the conserved cysteine residues (in *yellow*) within the 56 Pknbpxa sequences and the published Pknbpxa amino acid sequence [GenBank:ACJ54535]. Amino acid position with 3 variants is shaded in *red*. The amino acid positions are represented vertically

**Fig. 3:**
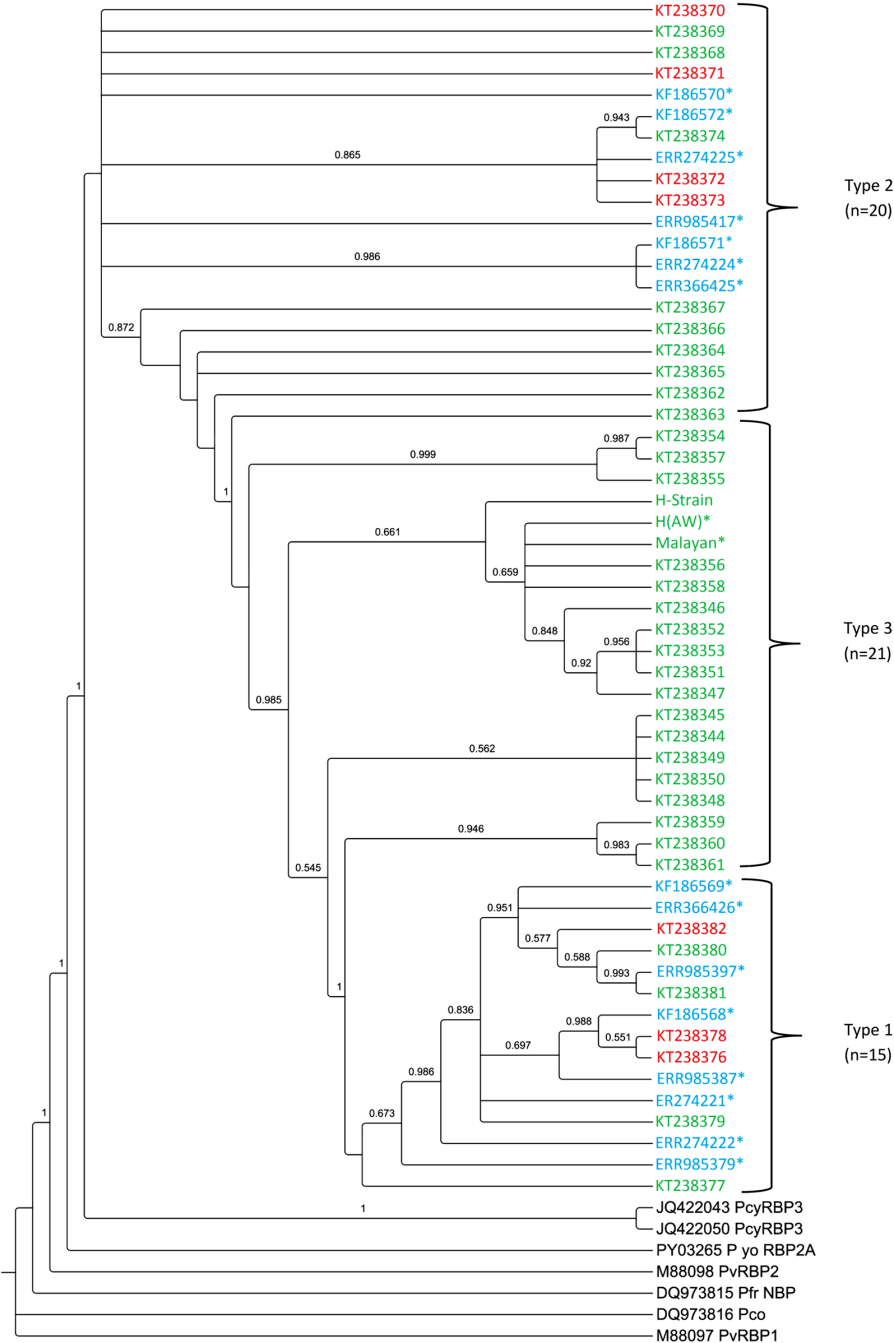
Bayesian phylogenetic tree of *Plasmodium* based on RBP gene sequences. The values above branches are posterior probabilities. Accession numbers in *red*, *green* and *blue* indicate samples originating from Sabah, Peninsular Malaysia and Sarawak respectively. RBP accession numbers in *black* are from of *Plasmodium vivax* RBP1 and 2, *Plasmodium yoelii* RBP2A, *Plasmodium cynomolgi* RBP3 which were used as out groups to draw the phylogeny. *Asterisk* indicates samples from previous study

**Fig. 4:**
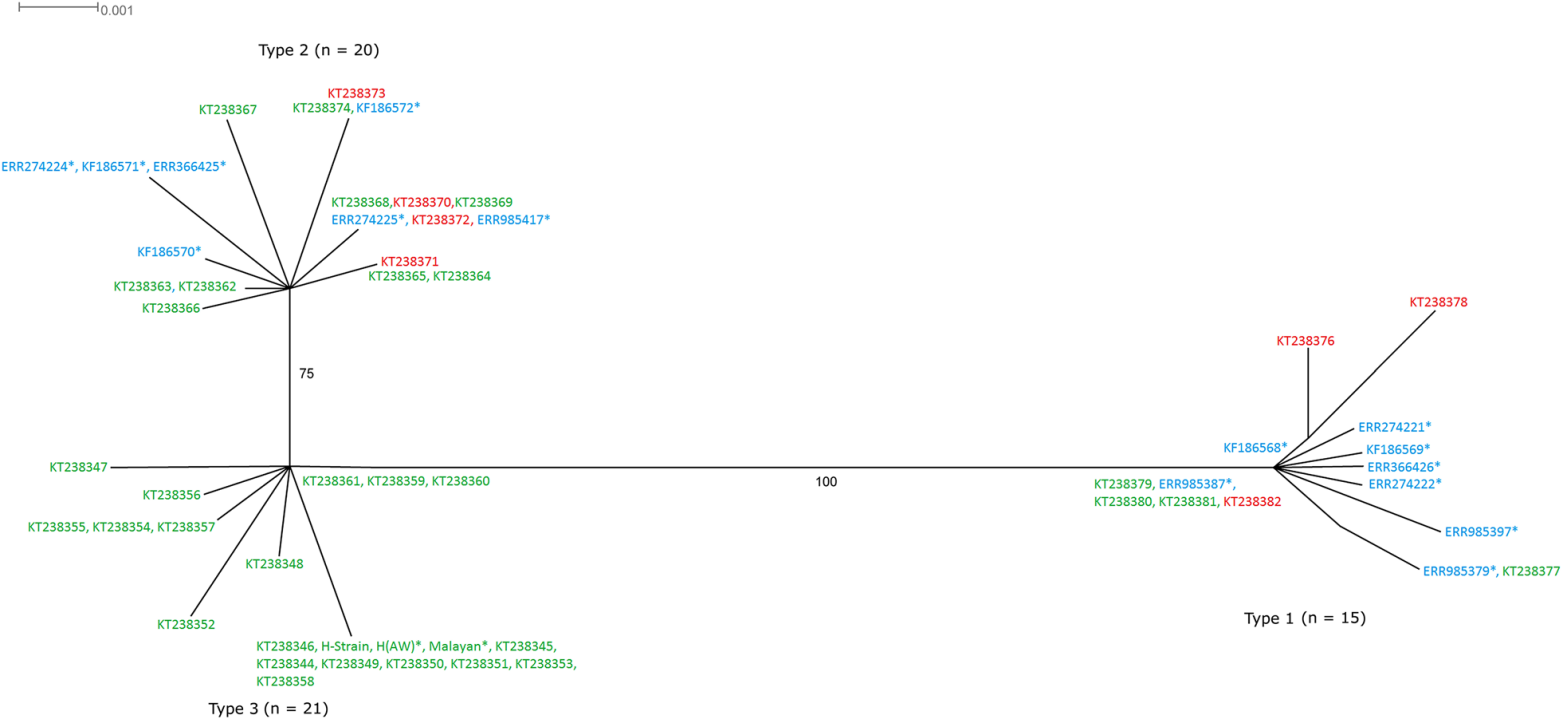
Split decomposition graphs of *Pknbpxa* sequences. The *graph* was constructed using the computer program SplitsTree (version 4.0). *Scale bars* show relative sequence dissimilarity. Analysis was run using 1000 bootstrap replications and only values above 70 are shown in the *graph*. Accession numbers in *red*, *green* and *blue* indicate samples originating from Sabah, Peninsular Malaysia and Sarawak respectively. *Asterisk* indicates samples from previous study

Of the 47 blood samples, high quality *Pknbpxa* gene (885 bp) sequences were obtained from only 38 samples [GenBank:KT238344-KT238382]. Four samples could not be amplified and five sequences were discarded as they produced unreadable sequencing results and two had mixed genotype infections. Together with the sequences retrieved from GenBank (mentioned in Methods), a total 50 *Pknbpxa* sequences were analysed. Multiple alignment of the *Pknbpxa* sequences revealed 75 (8.4 %) polymorphic and 810 (91 %) invariant sites (Fig. 1). Fifty-five (73 %) of the polymorphic sites were parsimony informative, of which 53 had two variants, and two had three variants (982; G/C/T and 994; A/C/G) (Fig. 1). Overall, there were 21 synonymous and 53 non-synonymous substitutions within the *Pknbpxa.* Dimorphism was evident in the alignment with 29 distinct SNPs changes observed within Type 1 and Type 2 (Fig. 1). Among these 29 SNPs, 25 (86 %) were non-synonymous and 4 (16 %) were synonymous substitutions. Five cysteine residues were found to be conserved within the sequences (Fig. 2). The average pairwise nucleotide diversity per site (π) for *Pknbpxa* was 0.02186. This nucleotide diversity level is higher than the diversity of some other *P. knowlesi* functional genes such as PkDBPαII (π = 0.012) [23], PkAMA-1 (π = 0.00501) [24] and PkRAP-1 (π = 0.01298) [25]. Phylogenetic trees constructed using Bayesian method showed that the sequences were divided according to Pknbpxa cluster types (Fig. 3), designated as Type 1 (n = 15), Type 2 (n = 20), and Type 3 (n = 21). Uniquely, the Type 3 cluster was observed to consist only of isolates originating from Peninsular Malaysia. The split decomposition graph of the 50 *Pknbpxa* sequences showed similarly separation of the types, with Type 2 and Type 3 being closely related (Fig. 4).

Codon based Z test for negative/purifying selection of Type 1 (n = 15) showed a significant result with dS − dN = 1.64 (p < 0.05). Types 2 and 3 (n = 41) also indicated negative selection but not significant, dS − dN = 0.452 (p > 0.32). The McDonald-Kreitman (MK) test also showed negative selection with significant departure from neutrality (NI = 0.341, p < 0.02) for Type 1. However, MK test for the Types 2 and 3 was not significant (NI = 0.71, p > 0.59).

Analysis of pairwise *F*_*ST*_ indicated high genetic differentiation among the types (*F*_*ST*_ > 0.66) (Table 1), with Type 1 and Type 2 having the highest level of differentiation (*F*_*ST*_ = 0.81).

**Table 1:** *F*_*ST*_ results for pairwise population comparisons and associated significance indications using Arlequin

Gene		Type 1 (n = 15)	Type 2 (n = 20)	Type 3 (n = 21)
*Pknbpxa*	Type 1 (n = 15)	–	**	**
	Type 2 (n = 20)	0.81	–	**
	Type 3 (n = 21)	0.71	0.66	–

** p < 0.001, p values computed with 10,100 permutations

## Discussion

A previous study has shown that the *Pknbpxa* could bind to human erythrocytes, suggesting that the protein may function as a ligand to enable the invasion of *P. knowlesi* merozoites into human cells [9]. The functional binding region of *Pknbpxa* was found to be dimorphic in clinical isolates of *P. knowlesi* from Sarawak [12]. The study the Sarawak isolates also showed that one of the *Pknbpxa* allelic forms (KH195 form) to be more virulent than the KH273 form, thus causing high parasitaemia and severe disease in some humans. Further investigation using next generation sequencing technologies discovered that this dimorphism extended throughout the *P. knowlesi* genome, and that two distinct types of *P. knowlesi* co-existed in Sarawak, Malaysian Borneo. Similarly, in this study, the *Pknbpxa* gene of *P. knowlesi* from Peninsular Malaysia and Sabah was found to have dimorphic forms. The core SNPs defining the two *P. knowlesi* types in natural infection as reported previously [12] were identified as well. The five conserved cysteine residues observed within the sequences indicated that ligand-receptor binding was intact within the three *P. knowlesi* types. Using Bayesian phylogeny approach, it was shown that Type 1 and Type 2 *Pknbpxa* were present in *P. knowlesi* from Peninsular Malaysia, Sabah and Sarawak. The Type 3 cluster, however, was found to contain samples *P. knowlesi* from Peninsular Malaysia only. Analysis using split decomposition graphs produced by distance-based network identified a well-supported bifurcation split (with 1000 bootstraps) between Type 1 and Types 2 and 3. A similar bifurcation was observed between the two non-recombining sub-species of *Plasmodium ovale* (*P. ovale curtisi* and *P. ovale wallikeri*) [26]. Therefore, there is also a possibility of the existence of two or more sub-species of *P. knowlesi* but this would require further genetic and morphological analysis. A third type of *Pknbpxa* was detected, consisting isolates originating only from Peninsular Malaysia. A recent study also reported a third cluster of *P. knowlesi* consisting of laboratory-adapted strains from Peninsular Malaysia [15]. However, it is likely that the laboratory adaptation process could be an explanation for this grouping rather than the geographic origin.

High genetic differentiation index (*F*_*ST*_ > 0.66) obtained between the *Pknbpxa* types. Recent genomic studies from Sarawak have also reported deep genetic differentiation [mean genome wide fixation index (*F*_*ST*_) = 0.21, with 9293 SNP having *F*_*ST*_ = 1] between the two clusters (sub populations) from clinical isolates [15]. The significant negative selection observed in *Pknbpxa* Type 1 is possibly the driving force in the evolution and separation of Type 1 from Type 2 and Type 3.

## Conclusions

This study revealed the existence of three *Pknbpxa* types in Malaysia. Types 1 and 2 were found not only in Malaysian Borneo (Sarawak and Sabah) but also in Peninsular Malaysia. A third type which was specific only to samples originating from Peninsular Malaysia was discovered. Further genetic studies with a larger sample size will be necessary to determine whether natural selection is driving this genetic differentiation and geographical separation.
